# Human Brucellosis in Febrile Patients Seeking Treatment at Remote Hospitals, Northeastern Kenya, 2014–2015

**DOI:** 10.3201/eid2212.160285

**Published:** 2016-12

**Authors:** John Njeru, Falk Melzer, Gamal Wareth, Hosny El-Adawy, Klaus Henning, Mathias W. Pletz, Regine Heller, Samuel Kariuki, Eric Fèvre, Heinrich Neubauer

**Affiliations:** Friedrich-Loeffler-Institut, Jena, Germany (J. Njeru, F. Melzer, G. Wareth, H. El*-*Adawy, K. Henning, H. Neubauer);; Friedrich Schiller University, Jena (J. Njeru, M.W. Pletz, R. Heller);; Kenya Medical Research Institute, Nairobi, Kenya (J. Njeru, S. Kariuki);; Benha University, Moshtohor, Egypt (G. Wareth);; Kafrelsheikh University, Kafr El-Sheikh, Egypt (H. El-Adawy);; University of Liverpool, Liverpool, United Kingdom (E. Fèvre);; International Livestock Research Institute, Nairobi (E. Fèvre)

**Keywords:** seroprevalence, *Brucella abortus*, brucellosis, bacteria, bacterial infection, fever, febrile, zoonotic, zoonoses, Garissa, Wajir, Kenya

## Abstract

During 2014–2015, patients in northeastern Kenya were assessed for brucellosis and characteristics that might help clinicians identify brucellosis. Among 146 confirmed brucellosis patients, 29 (20%) had negative serologic tests. No clinical feature was a good indicator of infection, which was associated with animal contact and drinking raw milk.

Brucellosis is a zoonotic disease that can cause severe illness in humans and substantial economic losses in livestock production (*1*). The main causative agents of brucellosis in humans are *Brucella abortus*, *B. melitensis*, and *B. suis* (*2*). Infection in humans occurs mainly by ingestion of contaminated animal products, inhalation of contaminated airborne particulates, or direct contact with infected animals or their products (*3*). Clinical signs and symptoms of human brucellosis are nonspecific and highly variable (*4*). Persons who work with animals and their families are considered to be at high risk for infection (*3*,*5*). In animals, brucellosis is asymptomatic but can cause abortions, weak offspring, and sterility (*5*).

In developing countries, serologic assays based on rapid slide agglutination tests are the mainstay for diagnosis of brucellosis, but these assays have poor specificity (*6*). Generally, ELISA is considered to be more specific and sensitive, allowing for a better correlation with the clinical situation. Although PCR assays are highly sensitive and specific tools for rapid diagnosis of human brucellosis and simultaneous differentiation of *Brucella* genotypes, they are often unavailable in many of these countries (*7*).

A review of brucellosis epidemiology in sub-Saharan Africa highlighted the fact that brucellosis is endemic in pastoral production systems where disease surveillance and control programs are poorly implemented (*1*). Within Kenya, seroprevalences of 2% and 7% have been reported among persons at high risk for brucellosis in Nairobi and Nakuru counties, respectively (*8*), and a national seroprevalence of 3% was reported in 2007 (*9*). More recently, Osoro et al. (*10*) showed variation (2.4%–46.5%) in seroprevalence across 3 counties in Kenya.

Diagnosis of febrile illnesses in developing countries is challenging because of the lack of imaging and reliable laboratory support. Clinical management of such illnesses is often done empirically, resulting in inaccurate treatment of patients and routine underreporting of disease (*11*). Data on the prevalence and potential risk factors associated with human brucellosis in Kenya are scant. The prevalent *Brucella* species in Kenya remain largely unknown. The purposes of this study were to assess the proportion of patients with brucellosis at 2 hospitals in northeastern Kenya and to describe patient characteristics that might help clinicians to identify brucellosis cases in areas without laboratory support.

## The Study

During 2014–2015, we enrolled patients with acute febrile illness seeking treatment at Garissa and Wajir hospitals in northeastern Kenya (Figure) by using systematic sampling intervals based on previously documented proportions of febrile patients recorded at each hospital. The study protocol was approved by the Scientific and Ethics Review Committee of Kenya Medical Research Institute. We obtained serum samples and tested them for brucellosis by using the modified Rose Bengal Plate Test (RBPT) (VLA Weybridge, United Kingdom) (*12*) and SERION ELISA classic *Brucella* IgM/IgG kits (Virion/Serion, Wurzburg, Germany) according to the manufacturers’ instructions. We extracted DNA from serum samples by using the High Pure Template Kit (Roche Diagnostics, Mannheim, Germany). We performed quantitative real-time PCR (qPCR) assays for the detection of brucellosis and speciation of *Brucella* species, as previously described (*13*) (Technical Appendix Table 1). We classified patients as having brucellosis if they had positive qPCR results or had positive RBPT results confirmed by positive ELISA results. We fitted multivariate logistic regression models to assess demographic, clinical features, and plausible risk factors associated with brucellosis seropositivity by using a stepwise backward analysis procedure.

**Figure:**
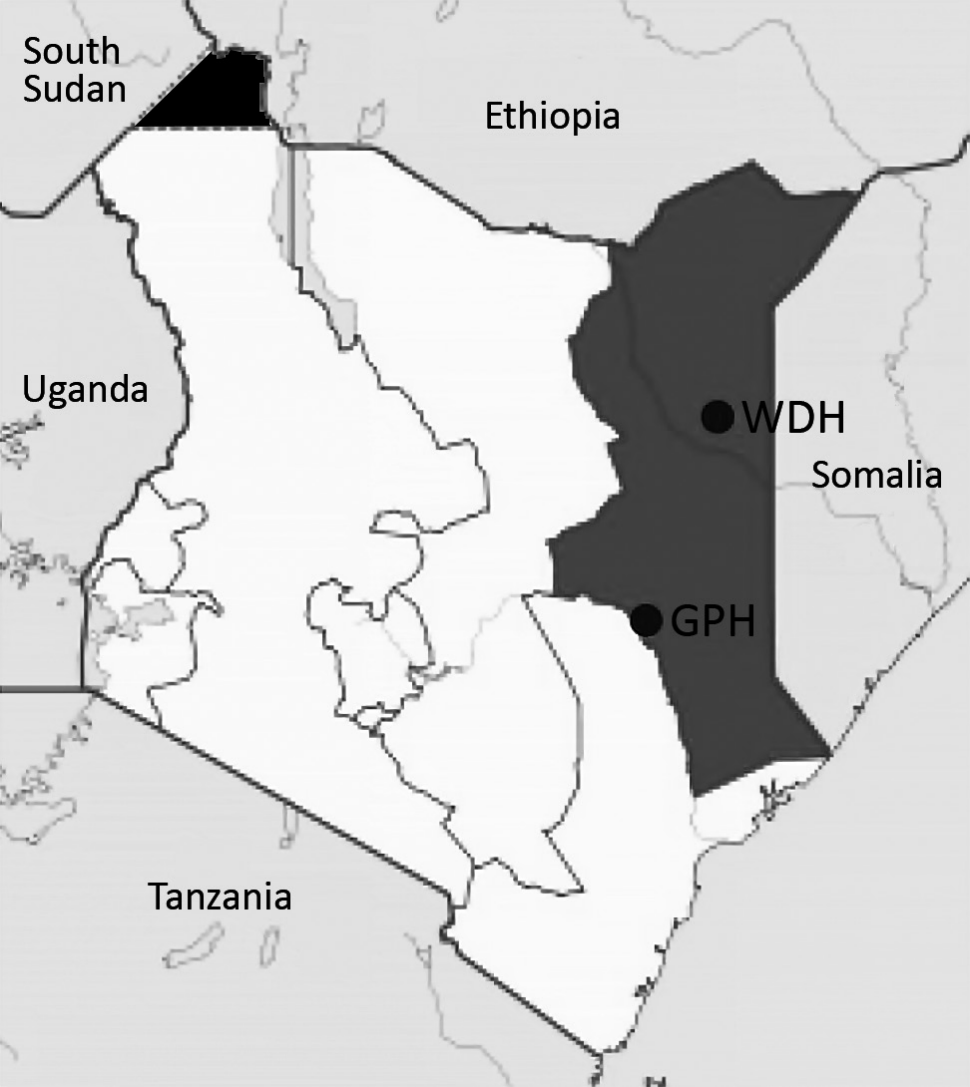
Locations of the 2 hospitals in the Northeastern Province of Kenya (dark gray shading) where human brucellosis was diagnosed in febrile patients seeking treatment, Kenya, 2014–2015. The solid black area in northwestern Kenya represents disputed territory among Kenya, Ethiopia, and South Sudan. GPH, Garissa Provincial Hospital; WDH, Wajir District Hospital.

Overall, 1,067 patients participated in the study; 580 (54.4%) of participants were female, and 963 (90.3%) were of Somali ethnicity (Technical Appendix Table 2). Brucellosis was established in 146 patients (13.7%, 95% CI 11.7%–15.9%). Of these, 29 (2.7%) had negative serologic test results for *Brucella* infection. *B. abortus* was the only *Brucella* species found using the *Brucella* species–specific qPCR.

Statistical analyses showed no significant differences in infection by ethnic group, county of residence, education status, or age group. Men had a significantly higher probability (odds ratio [OR] 1.98, p = 0.001) for having brucellosis (Table 1).

**Table 1:** Selected characteristics of study participants and number of febrile patients with *Brucella*-positive test results, northeastern Kenya, 2014–2015*

Characteristic	No. (%) patients		p value	Crude OR (95% CI)
	Positive for brucellosis, n = 146	Negative for brucellosis, n = 921		
Mean age, y, + SD	34.8 + 11.5	33.9 + 12.6	0.863	NA
Age group, y				
<19	16 (11.0)	127 (13.8)	NA	Referent
20–29	31 (21.2)	206 (22.4)	0.418	1.29 (0.70–2.39)
30–39	48 (32.9)	265 (28.8)	0.063	1.75 (0.97–3.08)
40–49	38 (26.0)	196 (21.3)	0.169	1.52 (0.84–2.76)
>50	13 (8.9)	127 (13.8)	0.764	0.90 (0.43–1.84)
Male sex	86 (58.9)	401 (43.5)	0.001	1.98 (1.30–2.68)
Wajir County resident	81 (55.5)	440 (49.4)	0.201	1.34 (0.97–1.77)
Occupation				
Herder	109 (74.7)	569 (61.8)	0.002	3.81 (2.17–12.38)
Civil servant	16 (11.0)	126 (13.7)	0.199	2.53 (0.71–8.91)
General business	7 (4.8)	67 (7.3)	0.286	1.98 (0.49–7.85)
Student	6 (4.1)	56 (6.1)	0.308	2.08 (0.51–8.48)
Livestock trader	6 (4.1)	53 (5.8)	0.053	3.74 (0.98–14.26)
Other	2 (1.4)	50 (5.4)	NA	Referent
Education level				
None	98 (67.1)	563 (61.1)	NA	Referent
Primary	25 (17.1)	181 (19.7)	0.335	0.79 (0.51–1.27)
Secondary	16 (11.0)	104 (11.3)	0.670	0.88 (0.69–1.56)
Post-secondary	7 (4.8)	73 (7.9	0.146	0.56 (0.25–1.32)
Somali ethnic group member	133 (91.1)	830 (90.1)	0.712	1.12 (0.62–2.63)
Clinical symptoms and signs				
Headache	113 (77.4)	836 (90.8)	0.504	1.45 (0.63–3.12)
Chills	93 (63.7)	482 (52.3)	0.065	1.79 (0.93–2.58)
Arthralgia/myalgia	118 (80.8)	699 (75.9)	0.322	1.48 (0.78–1.85)
Malaise/fatigue	101 (69.2)	646 (70.1)	0.018	2.20 (1.44–4.31)
Anorexia	63 (43.2)	514 (55.8)	0.610	0.91 (0.56–1.93)
Respiratory tract infection	34 (23.3)	263 (28.6)	0.434	1.03 (0.69–1.60)
Constipation	22 (15.1)	171 (18.6)	0.301	1.08 (0.83–3.11)
Night sweats	11 (7.5)	159 (17.3)	0.181	0.90 (0.67–5.90)
Diarrhea	8 (5.5)	106 (11.5)	0.120	0.95 (0.86–2.89)
Weight loss	12 (8.2)	105 (11.4)	0.228	1.24 (0.81- 6.04)
Confusion†	3 (2.3)	42 (5.3)	0.337	0.96 (0.60–2.91)
Rash	3 (2.1)	40 (4.3)	0.172	0.74 (0.22–1.55)
Vomiting	4 (2.7)	28 (3.0)	0.582	0.85 (0.36–1.98)
Abdominal pain	52 (35.6)	215 (23.3)	0.007	1.92 (1.35–5.64)
Hepatomegaly/splenomegaly	33 (22.6)	103 (11.1)	0.011	2.01 (1.63–8.10)
History of fever, >14 d	75 (51.4)	326 (35.3)	<0.001	3.71 (2.75–10.94)
Provisional diagnosis‡				
Typhoid fever	63 (43.2)	371 (45.0)	0.671	NA
Malaria	30 (20.5)	252 (30.5)	0.079	NA
Pneumonia	12 (8.2)	114 (13.8)	0.084	NA
Other§	14 (9.6)	Undefined	NA	NA
Days since fever onset/median	24.5/16	13.0/8	<0.001	NA


*NA, not available; OR, odds ratio. †Data available for adult and adolescent patients only. ‡includes clinical diagnosis made by attending hospital clinician. §Data not available for all patients.

Considerable low sensitivity levels were found for clinical diagnosis of brucellosis in both hospitals (Technical Appendix Table 3). Patients with brucellosis were mainly diagnosed with typhoid fever (63 patients [43.2%]), malaria (30 [20.5%]), pneumonia (12 [8.2%]), and other common tropical fevers or fevers of unknown origin (14 [9.6%]) (Table 1).

In the final combined multivariate analyses, brucellosis was significantly associated (p<0.05) with fever lasting >14 days (adjusted OR [aOR] 2.86), contact with cattle (aOR 6.50) or multiple animal species (aOR 2.35), slaughtering of animals (aOR 2.20), and consumption of raw cattle milk (aOR 3.88). Herders were 1.69-fold more likely to be seropositive (Table 2).

**Table 2:** Results of univariate and multivariate logistic regression analyses, by demographic, socioeconomic, and dietary risk factors associated with brucellosis, northeastern Kenya, 2014–2015*

Characteristic	No. (%) positive for brucellosis, n = 146	Crude OR (95% CI)	Adjusted OR (95% CI)†	p value
Occupation				
Herder	109 (16.1)	1.82 (1.22–2.71)‡	1.69 (1.25–3.44)	0.023
Other	37 (9.5)	Referent	Referent	NA
History of fever, >14 d				
Yes	75 (18.7)	3.71 (2.75–10.94)‡	2.86 (1.91–6.74)	0.003
No	71 (10.7)	Referent	Referent	NA
Contact with goats§				
Yes	107 (15.5)	1.31 (0.87–2.29)¶	Referent	NA
No	38 (10.1)	Referent	NA	NA
Contact with cattle				
Yes	101 (21.7)	3.15 (2.84–4.87)‡	6.50 (3.48–14.56)	<0.001
No	45 (7.5)	Referent	NA	NA
Contact with multiple animal species				
Yes	78 (19.6)	2.59 (2.16–7.66)‡	2.35 (2.14–8.63)	0.013
No	68 (10.2)	Referent	NA	NA
Frequent slaughtering of animals				
Yes	83 (21.7)	3.86 (3.21–5.69)‡	2.20 (2.07–5.87)	<0.001
No	63 (9.2)	Referent	Referent	NA
Frequent handling of raw milk				
Yes	93 (15.8)	1.41 (0.75–2.15)¶	NA	NA
No	53 (11.1)	Referent	Referent	NA
Frequent consumption of raw cattle milk				
Yes	86 (26.0)	4.07 (2.39–9.55)‡	3.88 (2.16–5.47)	<0.001
No	60 (8.2)	Referent	Referent	NA
Frequent consumption of locally fermented milk products				
Yes	72 (17.6)	1.68 (0.92–3.75)¶	NA	NA
No	74 (11.2)	Referent	Referent	NA
Frequent consumption of raw goat milk				
Yes	33 (17.9)	1.50 (0.98–2.95)¶	NA	NA
No	113 (12.8)	Referent	Referent	NA
Hosmer-Lemeshow goodness-of-fit test	NA	NA	NA	0.228
AUC (ROC)	NA	NA	0.745 (0.680–0.812)	<0.001

*AUC, area under the curve; NA, not available; OR, odds ratio; ROC, receiver operating characteristic. †Adjusted for age, sex, and site. ‡p<0.05. §Contact with goats (referent variable in multivariable model). ¶Variables with p<0.20 (Wald test) considered as potential risk and subsequently fitted in the multivariate analysis.

## Conclusions

This hospital-based study from a predominantly pastoral community in Kenya indicated a high prevalence (13.7%) of brucellosis in febrile patients, highlighting brucellosis as an important cause of acute febrile illnesses in northeastern Kenya. Although brucellosis has previously been described to occur in hospital patients in Kenya (*1*), it was not diagnosed by the treating hospital clinicians in 119/146 (81.5%) cases in our study. Instead, these cases were mainly attributed to other causes of fevers or fevers of unknown origin. In addition, 29 (2.7%) patients who had negative serologic test results for *Brucella* had positive results for *B. abortus* by qPCR. 

Our findings strongly suggest that patients with brucellosis were likely to leave the hospital without the specific treatment for brucellosis. This agrees with recent findings that showed that clinicians in Kenya continue to treat febrile patients for presumptive malaria, resulting in missed opportunities to accurately detect and treat other causes of fever (*11*,*14*). The results also highlight the usefulness of qPCR as a complementary assay to a combined ELISA and RBPT diagnostic approach in diagnosis of acute brucellosis and the need to establish national and regional reference laboratories with facilities for performing qPCR assays.

Contact with cattle or multiple animal species and consumption of raw milk from cattle were significantly associated with brucellosis in our study (Table 2). This association can be attributed to occupational and domestic contacts with livestock and social-cultural practices among communities in the study area that increase the risk for *Brucella* transmission, including nomadic movements, taking care of animals during parturition, consumption of raw milk from cattle and camels, and household slaughter of animals during traditional and religious ceremonies (*9*,*15*).

In this study, the only *Brucella* species detected was *B. abortus*, strengthening the assumption that brucellosis might be highly linked to cattle more than other animal species; however, further research is warranted. Additionally, the prevalent *Brucella* genotypes and biovars in Kenya remain to be determined.

Our study failed to better identify reliable clinical predictors for brucellosis. The lack of a clear clinical algorithm predictive of brucellosis supports the need for increasing clinician awareness of the disease and enhancing diagnostic capability for brucellosis in hospital settings.

This study has potential limitations. First, the study used acute-phase serum samples, making it difficult to demonstrate 4-fold titer rise. Follow-up of patients to obtain a convalescent-phase serum sample was not feasible because of ongoing inter-clan conflicts and militia activities in the region. Therefore, the possibility of patients who had previous exposure to *Brucella* but had residual antibodies in circulation cannot be ruled out.

Technical Appendix Primers and probes for *Brucella* genus and species-specific quantitative PCR on specimens from brucellosis patients, Wajir and Garissa hospitals, northeastern Kenya, 2014–2015. Characteristics of brucellosis patients. Sensitivity and specificity of clinical diagnosis compared with laboratory-confirmed diagnosis of brucellosis.
