# Supplementary material for: Human Brucellosis in Febrile Patients Seeking Treatment at Remote Hospitals, Northeastern Kenya, 2014–2015
Source: Emerg Infect Dis. 2016 Dec;22(12):2160–4. doi: 10.3201/eid2212.160285 (PMC5189133; doi:10.3201/eid2212.160285)
Supplement: Technical Appendix — Primers and probes for Brucella genus and species-specific quantitative PCR on specimens from brucellosis patients, Wajir and Garissa hospitals, northeastern Kenya, 2014–2015. Characteristics of brucellosis patients. Sensitivity and specificity of clinical diagnosis compared with laboratory-confirmed diagnosis of brucellosis. [file 16-0285-Techapp-s1.pdf]

Article DOI: http://dx.doi.org/10.3201/eid2212.160285

Human Brucellosis in Febrile Patients Seeking Treatment at Remote Hospitals, Northeastern Kenya, 2014–2015

Technical Appendix

Technical Appendix Table 1. Primers and probes for *Brucella* genus and species-specific qPCR on specimens from brucellosis patients, Wajir and Garissa hospitals, northeastern Kenya, 2014–2015

qPCR type	Forward primer (5′→3′)	Reverse primer (5′→3′)	Probe (5′→3′)
Brucella genus	GCTCGGTTGCCAATATCAATGC	GGGTAAAGCGTCGCCAGAAG	6FAM-AAATCTTCCACCT TGCCCTTGCCATCA-BHQ1
B. abortus	GCGGCTTTTCTATCACGGTATTC	CATGCGCTATGATCTGGTTACG	HEX-CGCTCATGCTCGCC AGACTTCAATG-BHQ1
B. melitensis	AACAAGCGGCACCCCTAAAA	CATGCGCTATGATCTGGTTACG	Cy5-CAGGAGTGTTTCGGCT CAGAATAACCACA-BHQ2

*qPCR, quantitative real-time PCR.

Technical Appendix Table 2. Select demographic, socioeconomic, and dietary characteristics of brucellosis patients, Wajir and Garissa hospitals, northeastern Kenya, 2014–2015

Characteristic	No. patients		Total no. patients (%)
	Garissa hospital	Wajir hospital	
Total	530	537	1067 (100)
Age group, y			
0–18	52	79	131 (12.3)
≥19	478	458	936 (87.7)
Sex			
Female	283	297	580 (54.4)
Male	247	240	487 (45.6)
Occupation			
Other	19	33	52 (4.9)
Livestock trader	34	25	59 (5.5)
Student	28	34	62 (5.8)
General business	44	30	74 (6.9)
Civil servant	73	69	142 (13.3)
Herder	332	346	678 (63.5)
Education level			
None	325	336	661 (61.9)
Primary	109	97	206 (19.3)
Secondary	57	63	120 (11.2)
Post-secondary	39	41	80 (7.5)
Ethnic group			
Somali	456	507	963 (90.3)
Other	74	30	104 (9.7)
Frequent contact with animal/products*			
Yes	428	457	885 (82.9)
No	102	80	182 (17.1)
Frequent consumption of raw cattle milk			
Yes	146	185	331 (31.0)
No	384	352	736 (69.0)
Frequent consumption of raw goat milk			
Yes	85	99	184 (17.2)
No	445	438	883 (82.8)
Frequent consumption of raw camel milk			
Yes	466	479	945 (88.4)
No	64	58	122 (11.4)
Frequent consumption of locally fermented milk			

Characteristic	No. patients Garissa hospital	Wajir hospital	Total no. patients (%)
Yes	194	215	409 (38.3)
No	336	322	658 (61.7)
Frequent consumption of animal blood			
Yes	16	29	45 (4.2)
No	514	508	1022 (95.8)
Frequent consumption of raw meat			
Yes	24	33	57 (5.3)
No	506	504	1010 (94.7)
Frequently prepare manure			
Yes	42	8	50 (4.7)
No	488	529	1017 (95.3)
Sharing house with animal			
Yes	62	36	98 (9.2)
No	468	501	969 (90.8)
Frequent contact with wildlife			
Yes	2	0	2 (0.2)
No	528	537	1065 (99.8)

*Defined as direct contact with livestock or contact with their products (e.g., meat, skin, and raw milk or other body fluids).

Technical Appendix Table 3. Sensitivity and specificity of clinical diagnosis compared with laboratory-confirmed diagnosis of brucellosis, Wajir and Garissa hospitals, northeastern Kenya, 2014–2015

Characteristic	% (95% CI) Garissa hospital	Wajir hospital	p value
Sensitivity	16.1 (9.15– 28.72)	19.8 (12.16–30.40)	0.590
Specificity	85.4 (81.83–88.49)	71.2 (67.25–74.97)	0.024
